# On the detection of cerebral metabolic depression in experimental traumatic brain injury using Chemical Exchange Saturation Transfer (CEST)-weighted MRI

**DOI:** 10.1038/s41598-017-19094-z

**Published:** 2018-01-12

**Authors:** Tsang-Wei Tu, Wael G. Ibrahim, Neekita Jikaria, Jeeva P. Munasinghe, Jaclyn A. Witko, Dima A. Hammoud, Joseph A. Frank

**Affiliations:** 1 Frank Laboratory, Radiology & Imaging Sciences, Clinical Center, National Institutes of Health, Bethesda, MD United States; 2 Center for Neuroscience and Regenerative Medicine, Henry Jackson Foundation, Bethesda, MD United States; 3 Molecular Imaging Laboratory, Department of Radiology, Howard University, Washington, DC United States; 4 Center for Infectious Disease Imaging, Radiology & Imaging Sciences, Clinical Center, National Institutes of Health, Bethesda, MD United States; 5 Acute Stroke Research Section, National Institute of Neurological Disorders and Stroke, National Institutes of Health, Bethesda, MD United States; 6 Mouse Imaging Facility, National Institute of Neurological Disorders and Stroke, National Institutes of Health, Bethesda, MD United States; 7 National Institute of Biomedical Imaging and Bioengineering, National Institutes of Health, Bethesda, MD United States

## Abstract

Metabolic abnormalities are commonly observed in traumatic brain injury (TBI) patients exhibiting long-term neurological deficits. This study investigated the feasibility and reproducibility of using chemical exchange saturation transfer (CEST) MRI to detect cerebral metabolic depression in experimental TBI. Phantom and *in vivo* CEST experiments were conducted at 9.4 Tesla to optimize the selective saturation for enhancing the endogenous contrast-weighting of the proton exchanges over the range of glucose proton chemical shifts (glucoCEST) in the resting rat brain. The optimized glucoCEST-weighted imaging was performed on a closed-head model of diffuse TBI in rats with 2-deoxy-D-[^14^C]-glucose (2DG) autoradiography validation. The results demonstrated that saturation duration of 1‒2 seconds at pulse powers 1.5‒2µT resulted in an improved contrast-to-noise ratio between the gray and white matter comparable to 2DG autoradiographs. The intrasubject (n = 4) and intersubject (n = 3) coefficient of variations for repeated glucoCEST acquisitions (n = 4) ranged between 8‒16%. Optimization for the TBI study revealed that glucoCEST-weighted images with 1.5μT power and 1 s saturation duration revealed the greatest changes in contrast before and after TBI, and positively correlated with 2DG autoradiograph (*r* = 0.78, p < 0.01, n = 6) observations. These results demonstrate that glucoCEST-weighted imaging may be useful in detecting metabolic abnormalities following TBI.

## Introduction

Cerebral metabolic abnormalities can be detected from weeks to years after injury^1–3^ as reported for many traumatic brain injury (TBI) patients^4–6^ as well as in animal studies^7–9^. Energy mismatch initiates neuro-metabolic cascades that could alter long-term cognition and hinder neurologic functions^2,6,10^. Prolonged decreases in metabolism may impair learning and memory^11–13^, cause progressive cerebral atrophy^14^ and place TBI patients at high risk for development of neurodegenerative diseases, such as dementia^15,16^ and Alzheimer’s disease^17,18^. Alterations in the cerebral glucose uptake and metabolism in TBI patients can be detected by 2 deoxy-2-(^18^F) fluoro-D-glucose (^18^F-FDG) positron emission tomography (PET) using ^18^F as a pharmaceutical tracer^19^. PET scans are limited by low resolution and the inability to perform longitudinal studies due to ionizing radiation exposure limits. Moreover, analysis of PET scans requires the incorporation of a lumped-constant that is dependent on arterial plasma glucose concentration. However, variations in glucose levels across the normoglycemic range over repeated measurements in TBI patients tend to obscure results^20–22^.

Recently, chemical exchange saturation transfer (CEST) magnetic resonance imaging (MRI) has been introduced as a sensitive, non-invasive technique with high spatial resolution that can detect various molecular species (e.g. glucose) via ^1^H–water ^1^H exchange without the need of radio-isotopes^23,24^. Multiple molecules, possessing exchangeable protons, can be evaluated at their respective chemical shifts by applying magnetization transfer (MT) saturation pulses over a range of frequency offsets across the water proton resonance frequency used as a reference (i.e. Z-spectrum)^25,26^. CEST MRI has been employed to evaluate glucose content, defined as glucoCEST. Contrast is generated by determining the asymmetry of MT ratio (MTR_asym_) on the Z-spectrum in a range of glucose chemical shifts upfield and downfield of the water proton resonant frequency^27^. GlucoCEST imaging following an intravenous (IV) bolus of glucose as contrast agent has been used to successfully distinguish colorectal tumor types in rats^28^ and characterize the perfusion-related properties and blood-brain barrier permeability in glioblastoma patients^29^. To date, the glucoCEST technique has not yet been utilized to explore changes of endogenous contrast within the resting brain.

The current study investigated the feasibility of using the glucoCEST-weighted imaging for detecting metabolic depression in the resting brain following TBI. A series of phantom experiments were performed to optimize the glucoCEST-weighted imaging parameters for glucose chemical shifts of 1.2, 2.1 and 2.9 part per million (ppm)^27,28,30^. Following the optimization of the CEST imaging parameters, we investigated the sensitivity and specificity of the image-contrast for assessing glucose concentrations. The glucoCEST-weighted imaging parameters were further evaluated in normal rat brains to determine the reproducibility and reliability of this approach over time. GlucoCEST-weighted imaging was then performed in a closed-head model of diffuse TBI in rats^31^. Results were compared to postmortem 2-deoxy-D-[^14^C]-glucose (2DG) autoradiography to determine changes of glucose uptake and metabolism before and after TBI. With appropriate optimization of the MT parameters, the glucoCEST-weighted imaging technique was reproducible and has the potential to detect cerebral metabolic abnormalities during *in vivo* studies.

## Results

### Phantom study

**Figure 1:**
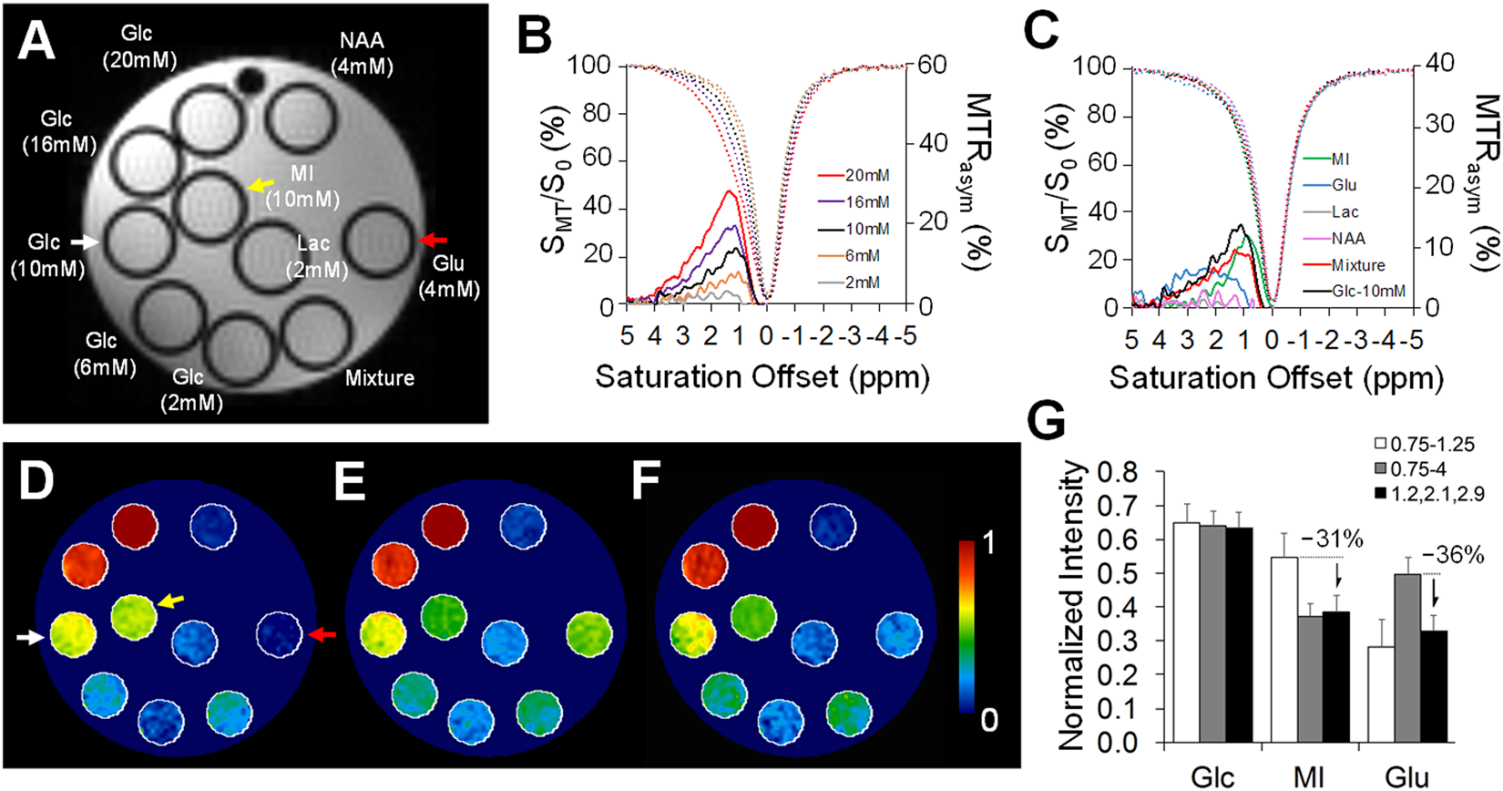
The CEST experiments on phantom metabolites consist of glucose (Glc, 2 to 20 mM), N-Acetylaspartic acid (NAA, 4 mM), myo-inositol (MI, 10 mM), lactate (Lac, 2 mM), glutamate (Glu, 4 mM) and a mixture of all metabolites (**A**). The Z-spectra and MTR_asym_ curves are displayed for glucose (**B**) and other metabolites (**C**). Comparisons between the derivations of the glucoCEST-weighted imaging were obtained from integrating the MTR_asym_ between 0.75‒1.25 ppm (**D**) and 0.75‒4.0 ppm (**E**) and 1.2 (±0.4), 2.1 (±0.2), 2.9 (±0.1) ppm (**F**). The quantification of Glc, MI, and Glu (arrows in **D**) shows that the current glucoCEST-weighted imaging improves the image contrast for glucose, while reducing ~30% contrast in MI and Glu. The representative glucoCEST-weighted images were acquired with a saturation power 5 uT and duration 2 s. The image intensity was normalized to its Glc (20 mM) intensity for comparison.

The phantom experimental results are shown in Fig. 1. The Z-spectra and MTR_asym_ curves obtained from the regions of interest (ROIs) in glucose (Glc) phantoms demonstrated the CEST effects from the Glc hydroxyl groups. Glc peaks were observable around 1.2, 2.1 and 2.9 ppm in the range of known biological concentrations (2–20 mM) (Fig. 1B). The Glc proton–water proton exchanges at the three resonances, however, were not clearly depicted because of the accelerated rate of exchanges between these pools of protons in the physiological pH level at 7.4^27^. Nevertheless, the MTR_asym_ curves were clearly distinguishable between different Glc levels of the range in biological concentrations (Fig. 1B). The other metabolites in the phantom had different exchangeable proton resonance offsets including myo-inositol (MI) 0.1 to 1.2 ppm; glutamate (Glu) with a broad coverage from 0.8 to 4ppm and a peak at 3ppm; and lactate (Lac) and N-Acetylaspartic acid (NAA) with negligible signals when employing the current acquisition parameters (Fig. 1C). Compared to the glucoCEST by integrating MTR_asym_ from 0.75‒1.25 ppm^32^ (Fig. 1D) and 0.75–4.0 ppm^28^ (Fig. 1E), the glucoCEST-weighted image derived from 1.2 (±0.4), 2.1 (±0.2) and 2.9 (±0.1) ppm (Fig. 1F) resulted in an approximate 30% decrease in signal intensity for MI and Glu (Fig. 1G), while still being able to maintain relevant contrast corresponding to the various Glc concentrations. A linear relationship (*r* > 0.9, *p* < 0.01, n = 5) was observed between the glucoCEST-weighted image contrast and the Glc concentration when MT saturation power was ≥1 μT (see Supplementary Fig. S1).

### Optimization of saturation amplitudes for *in vivo* brain imaging

**Figure 2:**
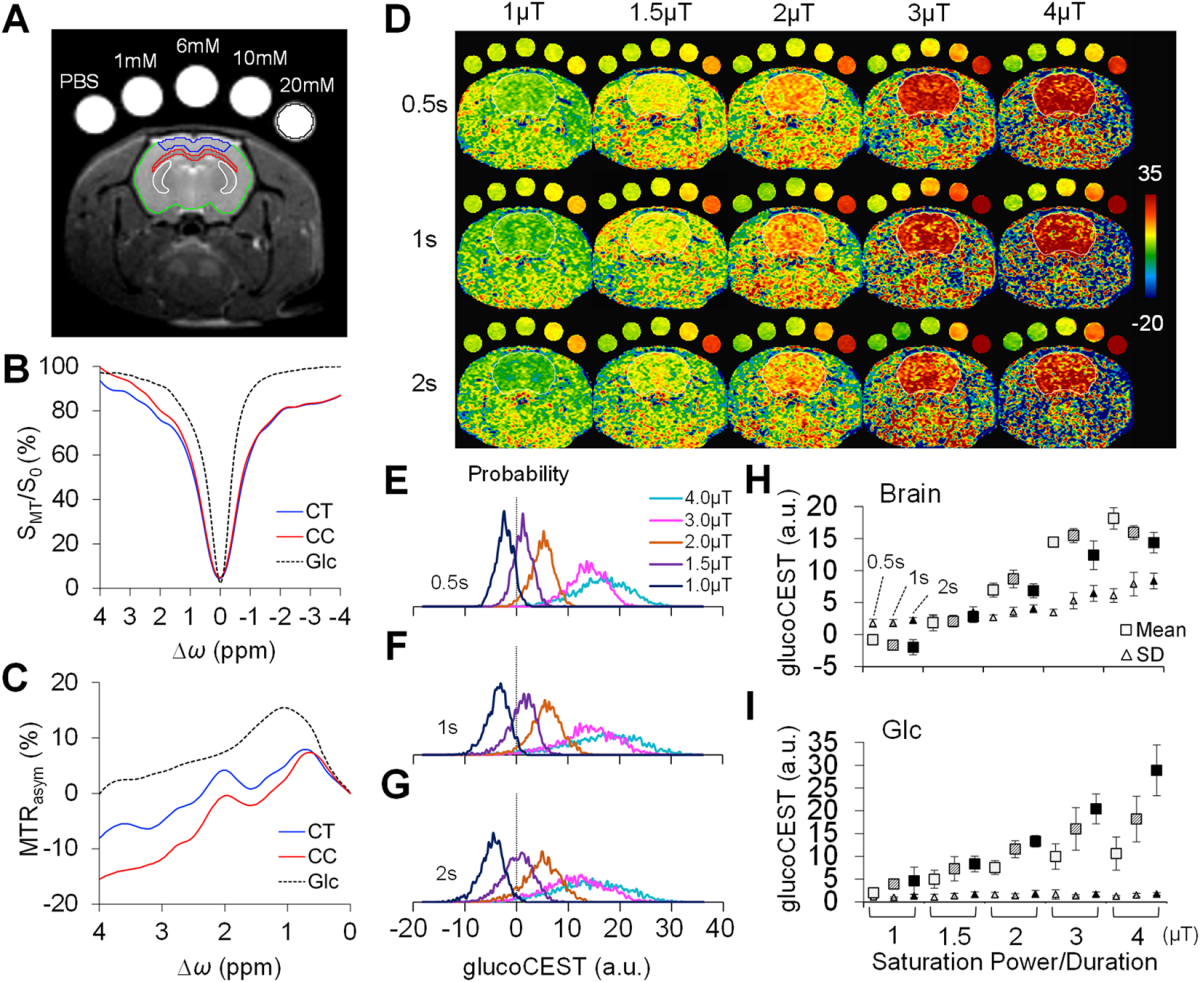
The setup for *in vivo* glucoCEST-weighted imaging on normal brains. Animals were imaged with five glucose phantoms consisting of 1 × phosphate-buffered saline (PBS), 1, 6, 10, 20 mM glucose (Glc) for comparison (**A**). The regions of interest denote the areas for data quantification of the cortex (CT, blue), corpus callosum (CC, red), caudate-putamen complex (Cpu, white, data used in Fig. 6), entire brain (green) and 20 mM glucose phantom (black). The Z-spectra (**B**) and the MTR_asym_ curves (**C**) acquired by 2 μT and 1 s saturation pulses showed CEST signals of Glc, CT and CC. The representative glucoCEST-weighted images were acquired using various saturation power and duration (**D**). While the glucoCEST-weighted images show clear contrast in response to different concentrations in phantoms, the glucoCEST images also differentiate contrast between CT and CC in the brain. The probability density of the brain glucoCEST signals is approximately Gaussian distributed for saturation duration of 0.5 s (**E**), 1 s (**F**) and 2 s (**G**). The averaged glucoCEST-weighted data in the brains (n = 6) show an increased mean and standard deviation (SD) by applying higher saturation power (**H**). In contrast, the signals in the 20 mM phantom increased in proportion to higher saturation power duration, while their SDs appear low across all experiments (**I**). (**H**) and (**I**) for each saturation power, points from the left to right denote the data acquired by 0.5 s (empty symbols), 1 s (pattern symbols), and 2 s (solid symbols) for saturation duration.

Figure 2 shows the *in vivo* glucoCEST-weighted imaging data obtained from rat brains. The averaged Z-spectra for the brains (n = 6) differed from the Glc phantoms due to interactions from radiofrequency (RF) irradiation effects and the presence of multiple metabolites in both cerebral cortex (CT) and corpus callosum (CC) (Fig. 2B). The Glc peaks in the *in vivo* MTR_asym_ profiles were not clearly apparent due to the effects of physiological pH level (~7.4) and overlapping exchangeable proton resonances from multiple metabolites in the brain (Fig. 2C). Figure 2D presents the series of *in vivo* glucoCEST-weighted images acquired from five Glc phantoms arranged in an array of saturation power and duration. Similar to the phantom data (Fig. 1 and Supplementary Fig. S2), the histograms of the brain glucoCEST appeared to have a Gaussian distribution (Fig. 2E–G). The kurtosis and skewness of the glucoCEST-weighted signals in the brain were not different throughout all evaluated saturation amplitudes, although there was an increasing deviation in kurtosis at 4 μT. While negative values appeared in the brain glucoCEST-weighted images at the saturation power of 1 μT, the intrasubject mean signal increased in proportion to the saturation power from 1 to 3 μT (Fig. 2H). No significant increases in glucoCEST-weighted signals were detected with increasing saturation powers from 3 to 4 μT. At all saturation powers, the glucoCEST-weighted signals using 2 s saturation pulses showed lower values when compared to those of shorter duration pulse of 0.5 s and 1 s. The intrasubject standard deviation (SD) of the glucoCEST-weighted signals increased significantly (*p* < 0.05, n = 6) when applying saturation powers ≥ 3μT to the brains. The SDs for the long saturation duration (2 s) also tended to increase as compared to the shorter duration (0.5 s) (Fig. 2H). In contrast, the signals in the 20 mM Glc phantom were consistently increased in proportional to both the saturation power and duration. However, the measured within-phantom SDs for all the tested saturation amplitudes were consistently low (Fig. 2I).

**Figure 3:**
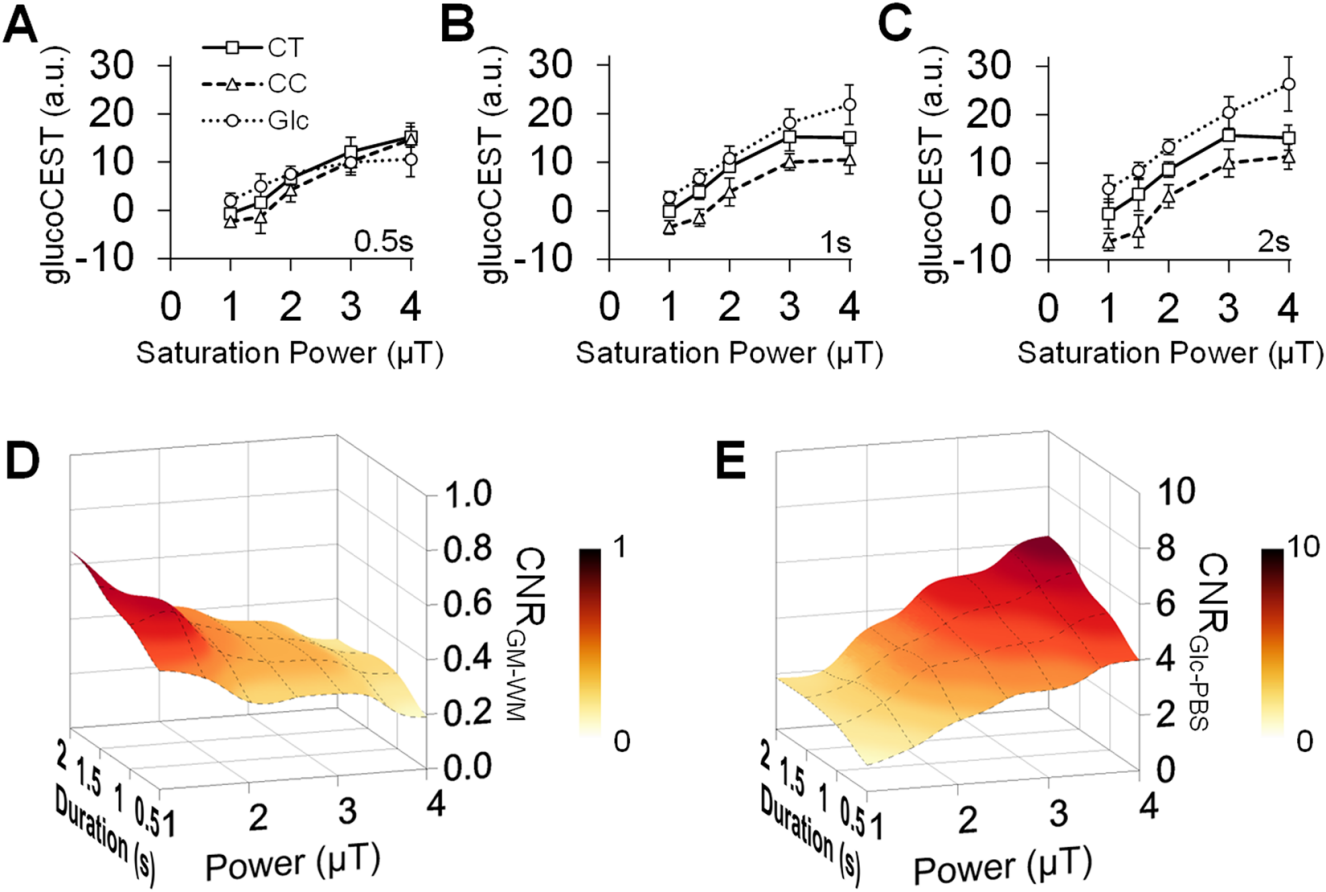
Comparison of the glucoCEST-weighted image contrast in brain and glucose phantom in response to multiple saturation amplitudes by saturation duration 0.5 s (**A**), 1 s (**B**) and 2 s (**C**). The resulting contrast-to-noise ratio (CNR) between the gray-white matter is maximized when applying saturation power ≤ 2μT and saturation duration 1–2 s (**D**). The CNRs in the Glc phantom increase with higher saturation power and longer saturation duration (**E**). CT: cortex, CC, corpus callosum, Glc: glucose, PBS: phosphate-buffered saline.

The group-averaged glucoCEST-weighted signals between subjects increased with the saturation power from 1 to 3 μT in the cortex (CT), while in the corpus callosum (CC) there was a significant (*p* < 0.05, n = 6) increase observed at saturation powers ≥ 1.5 μT (Fig. 3A‒C). For both CT and CC, the glucoCEST-weighted signals leveled out above a saturation power of 3 μT and saturation duration of 1 and 2 s (Fig. 3B,C). The contrast-to-noise ratio (CNR) between CT and CC decreased in proportional to the increased saturation power and the decreased duration (Fig. 3D). In comparison, the CNRs in the Glc phantoms continued to increase with increasing saturation powers and durations (Fig. 3E).

### Reproducibility of *in vivo* brain glucoCEST-weighted images

**Figure 4:**
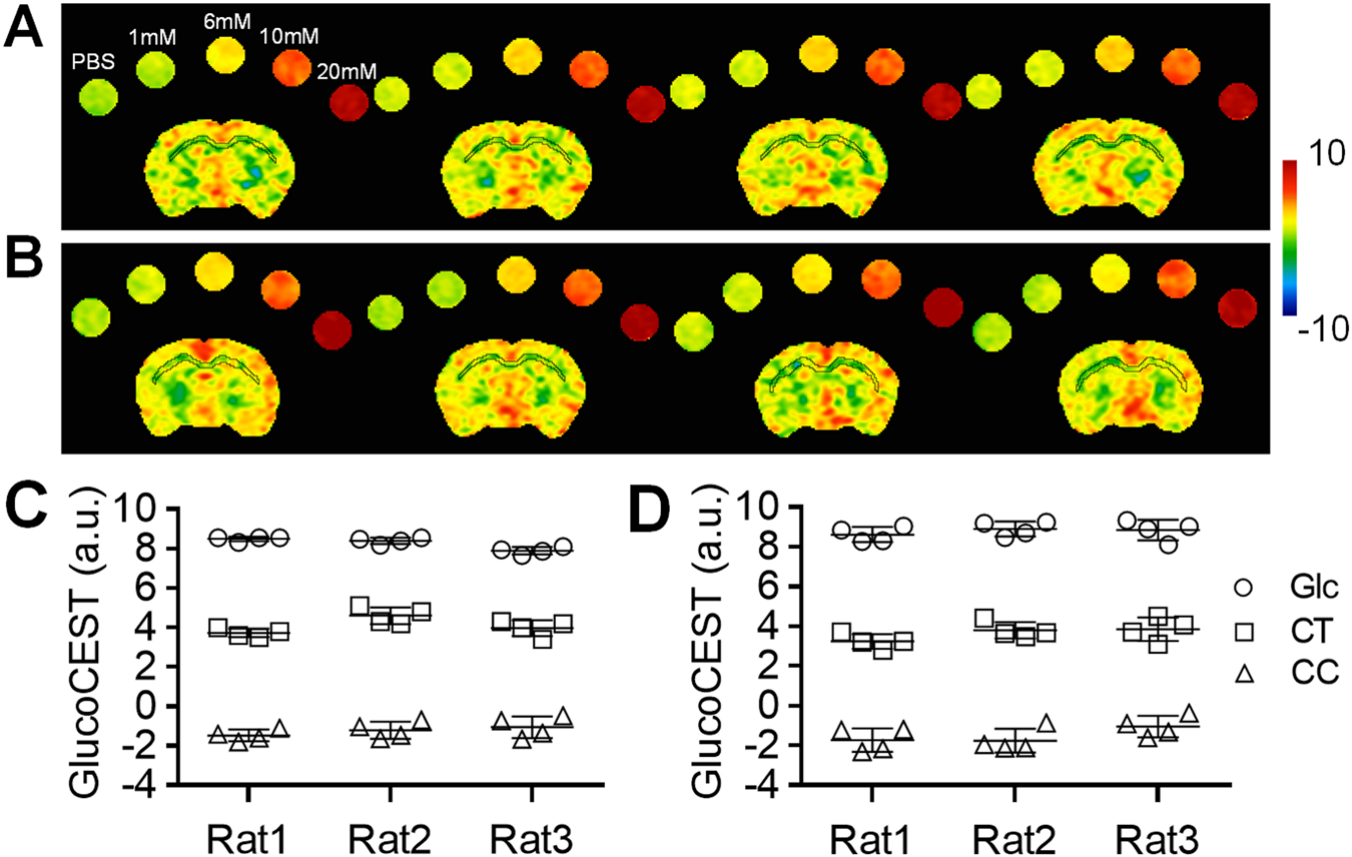
Representative glucoCEST-weighted images of repeating scans display consistent contrast in the brain from four continuous scans (**A**) and four weekly scans (**B**). The ROI quantification of the cortex shows an intrasubject coefficient of variation 8.4 ± 2.2% in the continuous scans (**C**) and 15.1 ± 2.9% in the weekly scans (**D**) (n = 3). CT: cortex, CC, corpus callosum, Glc: glucose, PBS: phosphate-buffered saline.

The *in vivo* brain glucoCEST-weighted images were reproducible across multiple scans (Fig. 4). The intrasubject coefficient of variation (CoV) was 8.4 ± 2.2% for the brain and 2.2 ± 0.6% for the Glc phantom in 4 continuous CEST experiments acquired within 2.5 hours (Fig. 4A). In comparison, when *in vivo* brain glucoCEST-weighted images were acquired weekly over 28 days, there was a CoV of 15.1 ± 2.9% for the brain and 7.2 ± 2.4% for the Glc phantom (Fig. 4B). The intersubject CoV was 16.4 ± 5.5% between animals and 7.8 ± 6.3% for the Glc phantoms. The averaged glucoCEST-weighted images showed significant differences (p < 0.01, n = 3) in values obtained from the CT (4.0 ± 0.3 a.u.) compared to the CC (−1.3 ± 0.2 a.u.) and 20 mM Glc phantom (8.3 ± 0.4 a.u.) (Fig. 4C,D).

### TBI study

**Figure 5:**
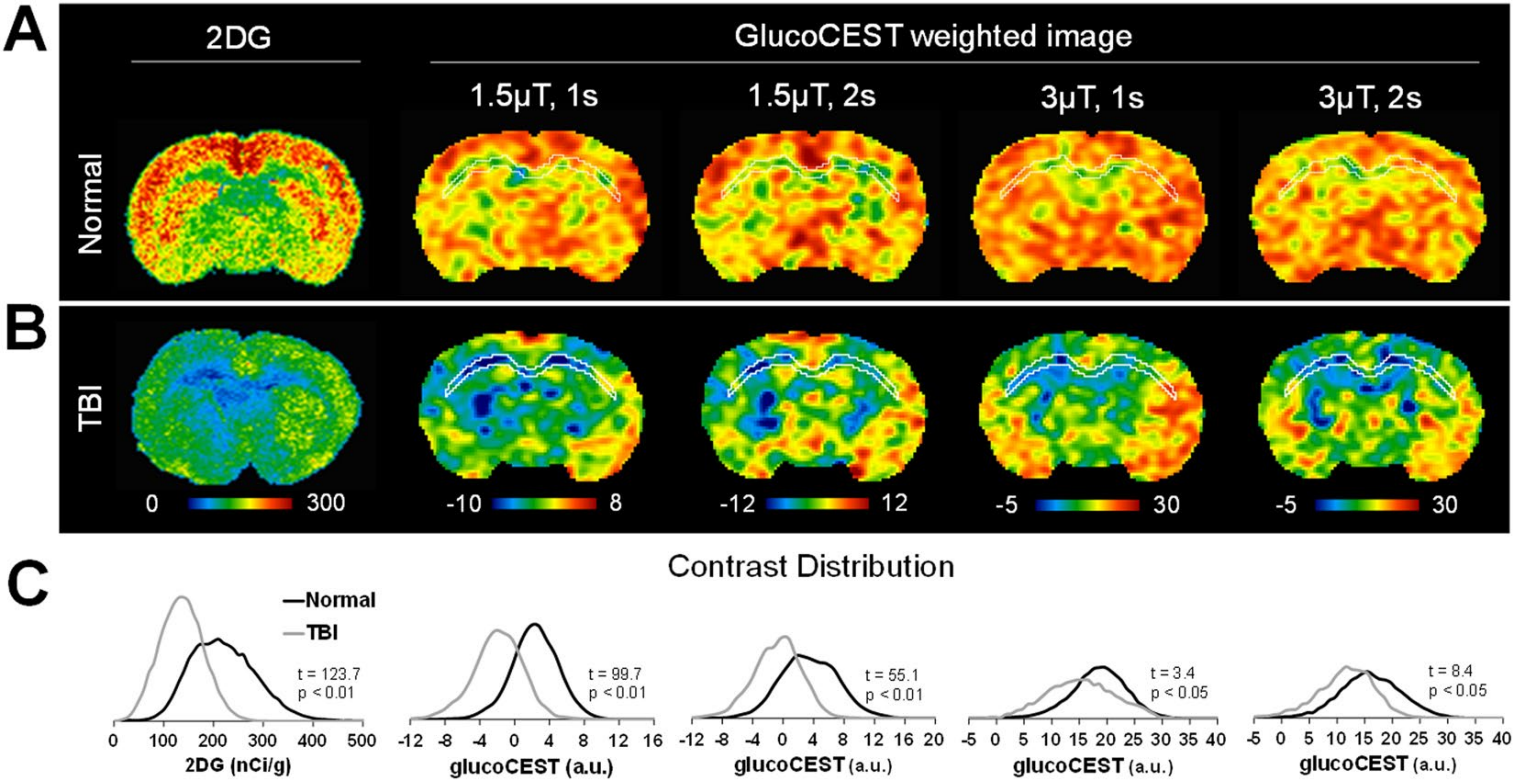
Comparison of the 2DG autoradiographs and glucoCEST-weighted images in the normal (**A**) and TBI brains (**B**). The images are scaled to mean ± 3 × standard deviation of the contrast in each group for cross comparison. The histograms of autoradiographs and glucoCEST-weighted images show the relative contrast shift between the normal and TBI brains (**C**).

TBI experiments were performed using a modified Marmarou weight drop closed-head injury model^31^. The 2DG autoradiographs displayed significant (*p* < 0.01, n = 3) difference in 2DG uptake in the CT (295.2 ± 43.8 nCi/g) and CC (129.2 ± 16.8 nCi/g) of the normal rats (Fig. 5A), with the intersubject CoV of 10.7%. Two weeks following TBI, autoradiographs clearly demonstrated decreases in 2DG uptake in the CT (186.0 ± 14.4 nCi/g) and CC (84.0 ± 10.6 nCi/g) (Fig. 5B,C). A wide variation in 2DG uptake was observed with significant (*p* < 0.01, n = 3) changes in the CC, CT, caudate-putamen complex (CPu) and thalamus in the injured brains. The glucoCEST-weighted images obtained with 1.5µT and <2 sec saturation pulses showed a clear pattern of decreasing contrast within the brains that was consistent with the 2DG observations (Fig. 5A,B). In comparison, the glucoCEST-weighted images performed at 3 μT demonstrated stronger RF irradiation effects especially in the ventral and lateral side of the brain at locations of greater field inhomogeneities. Histogram analysis of the glucoCEST-weighted images revealed that following TBI, the images exhibited significant decreases (t = 99.7, *p* < 0.01, n = 12) on the image obtained with a saturation power of 1.5μT and 1 s duration (Fig. 5C). Further analysis revealed that by acquiring glucoCEST-weighted images with saturation power of 3µT and 1 s duration, image contrast changes were reduced between the normal and TBI brains, yet the image contrast was still significantly different (t = 3.4, *p* < 0.05, n = 12).

**Figure 6:**
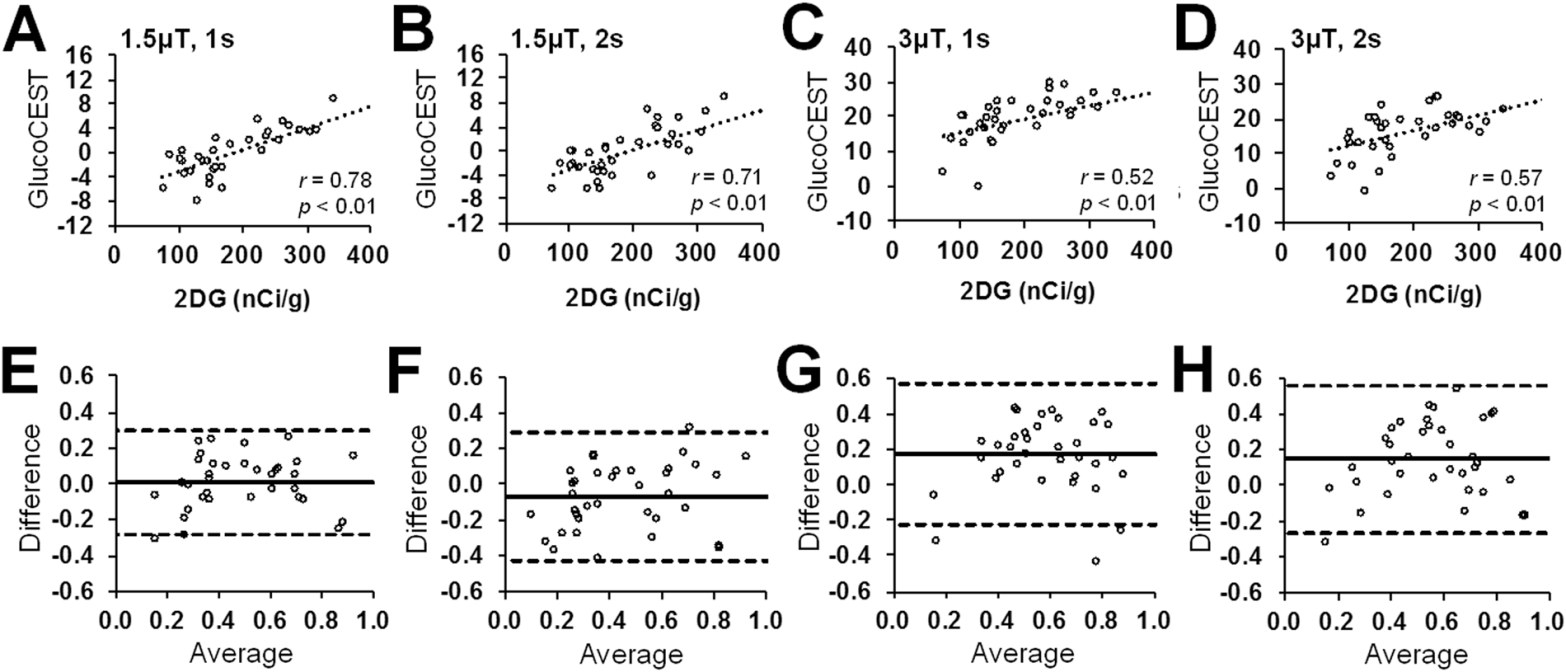
The correlations (**A**–**D**) and Bland-Altman plots (**E**–**H**) reveal the relationship and difference between the 2DG autoradiographs and the glucoCEST-weighted images acquired by different saturation pulses: 1.5 μT, 1 s (**A**,**E**), 1.5 μT, 2 s (**B**,**F**), 3 μT, 1 s (**C**,**G**) and 3 μT, 2 s (**D**,**H**). A higher correlation (*r* = 0.78, *p* < 0.01, n = 6) and agreement (95% CI: −0.28‒0.30) are seen between the autoradiographs and glucoCEST-weighted images by 1.5 μT, 1 s saturation pulses, where the 3 μT, 1 s saturation presents a lower level of correlation (*r* = 0.52, *p* < 0.01, n = 6) and agreement (95% CI: −0.23‒0.57) to the autoradiographs.

The correlation analysis revealed a moderate correlation (*r* = 0.52‒0.78, *p* < 0.01, n = 6) between the changes in autoradiographs and the glucoCEST-weighted images quantified from the external capsule, CC, CT and Cpu. (Fig. 6A–D). The corresponding Bland-Altman analysis showed that the glucoCEST-weighted images of 1.5μT saturation power and 1 s duration were in an agreement with a higher correlation level (*r* = 0.78, *p* < 0.01, n = 6) to 2DG autoradiographs (Fig. 6E,F). The 3 μT group exhibited a greater bias on the mean difference to the autoradiographic results (Fig. 6G,H).

## Discussion

The primary injury in diffuse TBI results initially in widespread parenchymal damage followed by secondary pathological processes. These include various physiological deficiencies, such as hypoxia, decreased blood flow, hyperglycemia and abnormal cerebral metabolism, which are associated with poor neurological outcome^6,33–35^. Invasive brain microdialysis techniques are widely used in monitoring brain glucose to detect the early signs of secondary injuries^36–38^. The crux of TBI patient care centers on the maintenance of adequate cerebral blood flow (CBF) in order to preserve tissues from irreparable damage caused by insufficient delivery of oxygen and glucose^20,39,40^. Likewise, insulin therapies have been evaluated in TBI patients as a method to regulate blood glucose in order to prevent further deterioration of brain tissue^41–43^. Thus, there is an increasing demand for a non-invasive imaging technique capable of evaluating glucose metabolism to inform the clinical and rehabilitative management of TBI patients^44,45^.

The current study describes a “proof-of-concept” investigation on the development, optimization and validation of glucoCEST-weighted imaging in a rat model of diffuse TBI. Following TBI, the glucose levels in the CT and CC were decreased by 46.9 ± 15.9% and 56.5 ± 13.6%, respectively, on 2DG autoradiographs, suggesting a hypometabolic state (Fig. 5). This hypometabolic phase in TBI is thought to be the consequence of a systematic decrease in CBF^46^, defects in glucose transporter function^13,47^, loss of neuronal integrity^48^ or decreased demand for glucose in tissues^10,49^. We observed comparable contrast changes with glucoCEST-weighted images following TBI (Fig. 5).

A recent study performed in head and neck cancer patients reported that glucoCEST-weighted imaging was able to differentiate between endogenous glucose content within the tumor and surrounding normal tissue^50^. Following an intravenous injection of glucose, it was shown that increased levels of glucose in the circulation enhanced the contrast differences between pre- and post-contrast glucoCEST-images within tissues. However, the high concentration of intravenous glucose needed to obtain such increases in image contrast may be detrimental to brain physiology by introducing alterations in CBF, osmotic pressure and membrane transport^50^. Meanwhile, glucoCEST contrast using saturation pulses of 1 μT and 5 s at 7 T have been effective in detecting glucose and glycogen in the kidney during the progression of diabetic nephropathy^51^. The current study demonstrates that glucoCEST-weighted images at 9.4 T could be optimized for endogenous contrast within brain with limited variability in repeated measures (Fig. 4). Acquiring glucoCEST-weighted images at high magnetic fields allows prolonged storage of saturation in the water pool, enhances the saturation efficiency and the frequency separation of hydroxyl protons by better adherence to the slow-exchange condition and reduces interference from direct water saturation^52–57^. Combined, these factors result in improved image contrast at high magnetic fields.

Optimizing image acquisition for *in vivo* CEST MRI was necessary in order to monitor ongoing pathological and physiological changes in normal and disease conditions. In the CEST Z-spectrum, the glucose hydroxyl groups resonate around 1.2, 2.1 and 2.9 ppm downfield from water with fast exchange rates of 500‒1500 s^−1^ ^27,28,30^. According to two-site exchange theory, these rapidly exchangeable protons can be saturated efficiently by applying high saturation power, however, extensive direct saturation (DS) effects can occur, decreasing sensitivity of glucoCEST in the brain^58^. The DS effect in combination with the solid pool macromolecular magnetization transfer contrast (MTC), and the intra/intermolecular nuclear overhauser enhancement (NOE) could also be amplified in the brain, thereby complicating the quantification of the CEST contrast at lower frequency separations from water^26,59^. Although the MTR_asym_ analysis could partially filter out the symmetric component of the DS effect, the asymmetric MTC and NOE from aliphatic protons of macromolecules/metabolites still exist in the brain. These compete with glucose in the same chemical shift range of desired proton exchange. Therefore, more critical RF irradiation levels and validation studies will be required for *in vivo* brain glucoCEST-weighed imaging to detect metabolic abnormalities.

In the current study, the RF irradiation level for *in vivo* glucoCEST was optimized based on the comparison of results obtained with 2DG autoradiography in the rat brain (Figs 5 and 6). 2DG is a glucose molecule whose 2-hydroxyl group is replaced by hydrogen so that glycolysis cannot proceed after phosphorylation to 2-deoxy-D-glucose-6-phosphate (2DG6P) by hexokinase. It serves as a good marker for glucose uptake and hexokinase activity in brain parenchyma^60^. In our experiments, 2DG was injected 45 minutes prior to euthanasia in order to reach a steady-state between glucose supply and consumption within the brain. The level of glucose uptake was clearly distinguishable between gray and white matter by 2DG (Fig. 5). High levels of glucose uptake were observed in the CT and CPu on autoradiographs^60^, whereas lower optical density was detected in the CC and ventral striatum (around half of that observed in CT and CPu^61,62^). This pattern was also seen in the glucoCEST-weighted images acquired with low saturation amplitude (Fig. 5A), although few discrepancies appeared in the ventral and lateral side of the brain that might be associated with greater B_0_ field inhomogeneities.

The *in vivo* glucoCEST signals from the rat brain were approximately Gaussian distributed (Fig. 2E–H), suggesting that the sequence parameter set was sensitive to random proton exchange processes in the parenchyma. However, when applying saturation powers > 3 μT, the variance of the signal distributions increased, reducing the CNR between CT and CC due to the excessive DS effect on the water spillover to the surround proton resonances. Applying a saturation power < 1 μT resulted in low-labeling efficiency of the hydroxyl protons which caused an attenuated CEST effect such that the MTC and NOE effects become dominant and generate a negative MTR_asym_. In contrast to the prolonged saturation duration (4.8‒6 s) in previous dynamic glucoCEST enhancement (DGE) studies^28,32^, we observed that a saturation duration ≤ 2 s was needed for the glucoCEST-weighted imaging to observe the contrast between gray and white matter at 9.4 T. In TBI brains, there was higher correlation observed between the 2DG and glucoCEST-weighted images acquired using low saturation power (1.5‒2μT) and short saturation duration (1‒2 s), even in the presence of MTC and NOE effects (Fig. 6).

Validating the origin of the glucoCEST contrast is crucial for monitoring changes associated with pathology. A recent study investigated the source of the dynamic glucoCEST contrast by coupling the infusion of glucose and CBF measures by MRI along with NMR spectroscopy^32^. This study showed that the dynamic glucose-enhanced signals of glucoCEST in rat brains were not affected by cerebral or plasma pH nor correlated with hypercapnia-induced alterations of CBF. The glucoCEST signals were related to the intracellular concentration of glucose by comparing the dynamic contrast difference between the D- and L-glucose infusions since L-glucose does not enter cells. By correlating *in vivo* ^31^P NMR measurements of infused 2DG6P in the rat with glucoCEST signals, the study suggested that the glucoCEST signals could be related to glucose uptake and metabolism.

Endogenous glucoCEST contrast in the brain has not been well understood. Presumably, endogenous glucoCEST contrast is related to steady-state glucose and other intracellular metabolites that resonate between 0.75‒2.9 ppm. However, the overlapping signals from multiple metabolites, such as MI, that resonate between 0.1‒1.2 ppm confound the measurement of glucose on glucoCEST-weighted images. In the current study, the glucoCEST-weighted images were derived by focusing on the glucose proton exchanges and narrowing the integration of MTR_asym_ at glucose chemical shifts, thereby reducing the interference from other metabolites (Fig. 1D‒F). Following TBI, cerebral MI significantly increases as detected by ^1^H-NMR spectroscopy studies^63–65^ and has been attributed to increased astrogliosis^31,66^, whereas glucose uptake is known to decrease^2,4,7,8^. In the current study, we were able to show that endogenous glucoCEST contrast was decreased following TBI and correlated to the findings on 2DG autoradiography. Further investigation is required to determine if the contribution of MI signal “subtracts” or “adds” contrast to glucoCEST-weighted imaging in TBI.

Limitations of this study need to be discussed to avoid misinterpretation of the results. First, the current study did not survey a complete neuro-metabolic profile in the TBI brains to determine the contribution from other metabolites (e.g. glutamate, NAA, lactate or MI) to glucoCEST contrast. The correlation between glucoCEST and 2DG autoradiography does not exclude the possibility that other metabolites might also contribute to changes in image contrast following TBI. Secondly, relating glucoCEST contrast to 2DG autoradiographs is not straightforward. In theory, the 2DG autoradiography measures the metabolic rate of glucose by acquiring the signal from the intracellular 2DG6P that is accumulated after 45 min of metabolism and washout. The endogenous glucoCEST signal, however, may represent total glucose from the sum of intra- and extra-cellular space, which requires further investigation. Meanwhile, the glucoCEST-weighted imaging was acquired on the rats under anesthesia, whereas the 2DG autoradiography was acquired in awake rats for a 45 min circulation time. Although imaging and 2DG results both observed metabolic abnormalities in TBI brains, the level of glucose metabolism might be underestimated by glucoCEST imaging^67^. In addition, there were significant partial volume effects when comparing 800 µm thick CEST images to 20 µm 2DG autoradiography sections. It is also important to note that the Glc peaks may not be apparent in several conditions, including high glucose concentration, pH > 6.2, or under multiple complications from other metabolites in the living tissue^27^. The addition of advanced acquisition or post-processing techniques, such as the multi-pool proton exchange model^68,69^, Lorentzian decomposition of the CEST Z-spectra^70,71^ or on-resonance spin-lock techniques^72,73^ might provide mechanisms to better isolate glucose resonances from other metabolites.

Previous studies have reported high reproducibility (CoV < 3%) in repeated CEST experiments^74–76^, but unfortunately the demonstration of reproducibility between laboratories of CEST MRI is difficult due to strong dependence on the saturation efficiency in each experimental setting^26^. The optimized parameters in the current study may not be applicable to other MR systems using different imaging parameters (e.g., volume, surface or phase array RF coils, etc.). Other factors including the temperature, proton exchange-rate in living tissue, pH level, T_1_, T_2_, the location of a subject within RF coil and RF pulse amplitude may also affect the contrast generated on CEST images^77^. Standardized procedures for optimization must be established for the CEST imaging parameters to ensure reproducible results^58^. Future studies should also include pathological, spectroscopic or molecular investigations to increase specificity for endogenous glucose detection in normal and injured brains, and examine how they compare to the observed glucoCEST-weighted images.

## Methods

### Phantom study

Phantom experiments were conducted to characterize the Z-spectrum and MTR_asym_ of glucose by a Doty quadrature coil (Doty Scientific, Inc., Columbia, SC) in a Bruker 9.4 T scanner (Bruker Corp., Billerica, MA). The phantom consisted of five glucose concentrations mimicking *in vivo* brain levels (2‒20 mM)^78^, together with the other common brain metabolites in biological concentrations, including NAA (4 mM), MI (10 mM), Lac (2 mM), Glu (4 mM)^79^ and a tube containing a mixture of these metabolites (Fig. 1A). The metabolite tubes were immersed in the 1 × phosphate-buffered saline (PBS) and all the solutions were corrected to the physiological pH 7.4. The magnetic field (B_0_) experienced within a volume of 30 × 30 × 5 (mm^3^) was made as homogenous as possible by 1^st^ and 2^nd^ order optimization of shimming (half height line width of <10 Hz). The CEST data were acquired by 2D Rapid Acquisition with Relaxation Enhancement (RARE) sequence with (S_MT_) and without (S_M0_) MT preparation pulses by repetition time (TR) 3.10 s, echo time (TE) 10.39 ms; RARE factor 4; number of excitation (NEX) 1; in plane resolution 400 μm^2^, slice thickness 800 µm. The effect of MT pulse amplitudes on the sensitivity and specificity for glucose detection were inspected by arraying the saturation power from 0.5 to 10μT and saturation duration from 0.5 to 3 s. The Z-spectrum was acquired from the MT frequency offsets (Δω) from −2 to +2 kHz with 50 Hz frequency stepping, as a result, 81 points were sampled to delineate the Z-spectrum from −5 to +5ppm.

### Data processing for the glucoCEST-weighted image

Procedure according to the WAter Saturation Shift Referencing (WASSR) method^80^ was applied to correct the B_0_ and B_1_ field inhomogeneity of the CEST data. In brief, a separate WASSR dataset was acquired with small saturation amplitude (0.2μT for power and 0.1 s for duration) from −0.4 kHz to + 0.4 kHz with 10 Hz frequency stepping to obtain the B_0_ field map. The CEST Z-spectrum was interpolated to 0.1ppm stepping and fitted to Lorentzian line shapes in combination with WASSR data to correct the shifted water resonance frequency by B_0_ field inhomogeneity. The B_1_ field map was obtained by the same RARE sequence with two flip angle (30° and 60°) and the pixel-wise B_1_ values were calculated by solving the equation [1]:

1 $$\frac{\cos \,2\theta }{\cos \,\theta }=\frac{S(2\theta )}{S(\theta )},$$

where *S*(*θ*) and *S*(2*θ*) are the pixel signals in an image with preparation flip angle *θ* and 2*θ* respectively. The B_1_ field map was obtained by *B*_1_ = *θ*(360*τ*)^−1^. The coefficient B_1_/B_1ref_ was used for B_1_ correction of glucoCEST contrast, where B_1ref_ was the reference images for RF pulse amplitude of a 30° flip angle. After correction, the robust CEST parameter, the MTR_asym_ was derived by^74,79^:

2 $$MT{R}_{asym}({\rm{\Delta }}\omega )=\frac{{S}_{MT}(-{\rm{\Delta }}{\rm{\omega }})-{S}_{MT}({\rm{\Delta }}{\rm{\omega }})}{{S}_{MT}(-{\rm{\Delta }}\omega )},$$

where *S*_*MT*_(−Δ*ω*) and *S*_*MT*_(Δ*ω*) are respectively the upfield and downfield MT signal intensity around the water resonance frequency at 0 Hz. The pixel-by-pixel signals of the glucoCEST-weighted image were generated by integrating the area under the MTR_asym_ curves at the glucose chemical shifts in 1.2 (±0.4), 2.1 (±0.2) and 2.9 (±0.1) ppm^28,30^ with an arbitrary unit (a.u.). Results were compared to the previous derivation definition of glucoCEST using 0.75‒1.25 ppm^32^ and 0.75‒4.0 ppm^28^ for showing the effect of reducing interactions from other metabolites.

### Rat model of diffuse TBI

All animal studies were approved by the animal care and use committee at the National Institutes of Health, and experiments were performed according to the National Research Council’s Guide for the Care and Use of Laboratory^81^. Female 8-week-old Sprague-Dawley rats (Taconic, Hudson, NY) were used in the TBI study. Rats first underwent baseline T2-weighted (T2W) images by RARE (TR 3.8 s, TE 15 ms, RARE factor 8, in-plane resolution 100 μm^2^ with 500 µm slice thickness) according to a previously published screening guideline to exclude the complication of spontaneous ventriculomegaly or impaired circulation system that might affect the perfusion and glucose metabolism^82^. Eight rats were identified as having normal morphology and treated with a modified version of the Marmarou weight drop closed-head injury for diffuse TBI as previously described^31^. Another six rats with normal morphology were served as the control group and used for the reproducibility experiments.

### *In vivo* MRI

Imaging was conducted two weeks after TBI to evaluate the changes in cerebral glucose uptake between the normal controls and TBI animals. Animals were anesthetized using an isoflurane/oxygen mixture via nosecone. The isoflurane level was maintained minimum (0.7–1.5%) for the MRI, right above the level that the animals moved. The body temperature was maintained at 37 °C using a circulating water bath; a steady respiration rate (47 ± 3 bpm) was maintained by anesthesia control. Three-dimensional T_2_*-weighted images were acquired for examining the presence of hemorrhage by multiple gradient echo: TR 60 ms, TE 3.18 ms, echo spacing 3.25 ms, voxel size 200 μm^3^ (isotropic). 2 rats were found with hemorrhage and they were excluded in the subsequent TBI study to eliminate the complication of hemorrhage in deviating the local field homogeneity for an accurate MTR_asym_ estimation. Glass capillaries filled with 1 × PBS and four concentrations of glucose solution (1, 6, 10 and 20 mM) were placed equidistantly above the rat head and imaged with the brain for comparison (Fig. 2A). 2^nd^ order Mapshim was applied to cover the brain in a 16.5 × 14.1 × 6.5 (mm) FOV to reach 40‒50 Hz line widths for field homogeneity. The CEST data were acquired using the same imaging setting in the phantom experiment by RARE: TR 2.06 s, TE 10.39 ms; RARE factor 4; NEX 1; in plane resolution 354 μm^2^, slice thickness 0.8 mm. An optimization study was conducted to determine the ideal combination of MT saturation power and duration for enhancing the detection of endogenous glucose in the rat brain. MT preparation pulses were arrayed for saturation power from 1, 1.5, 2, 3 and 4 μT, and saturation duration from 0.5, 1 and 2 s. The Z-spectrum was acquired from −1.6 to +1.6 kHz with 100 Hz frequency stepping to sample 33 points covering the frequency offset range from −4 to +4 ppm. The WASSR data for the rat brain were collected by the same RARE parameters with small saturation amplitude: power 0.3 μT, duration 0.25 s, frequency offset −0.4 to +0.4 kHz with 80 Hz frequency stepping. The *in vivo* Z-spectra were corrected for the B_0_/B_1_ field inhomogeneity to derive the MTR_asym_ curves and the glucoCEST-weighted images of the brains. The reproducibility of the *in vivo* brain glucoCEST was conducted on 3 normal rats by comparing 4 continuous acquisitions repeated in 2.5 hours, and 4 weekly acquisitions over 28 days with saturation power 1.5 μT, duration 1 s, and NEX 2.

### 2-deoxy-D-[^14^C]-glucose (2DG) autoradiography

After MRI, three rats per group were randomly picked for 2DG autoradiography. Rats were anesthetized with isoflurane (3% with 700cc/min O_2_) and [^14^C]−2DG (American Radiolabeled Chemicals Inc., St Louis, MO) was delivered as an intravenous bolus injection of 125µCi/kg body weight via the tail vein. Animals were awake for a tracer circulation time of 45 minutes, and then euthanized by isoflurane overdose, decapitated and brains were rapidly removed. The brains were then submerged shortly in 0.3 M sucrose on the ice, sectioned into 3 mm sections, frozen on dry ice and stored at −80 °C. Blocks were cut in a cryostat at −15 °C in 20 μm thick coronal sections then dried on glass slides. Slides were covered with scintillating sheets and exposed for 18 hours together with [^14^C] standard (ARC-146C(PL); St. Louis, MO) then analyzed in Microimager (Biospace lab, France). Measurements were performed on selected regions of interest (ROIs) placed in gray matter from CT and white matter in CC. Counts obtained from each slide were calibrated on the [^14^C] standard and values were then converted to nCi/g tissue.

### Imaging and 2DG data analysis

The Z-spectra, MTR_asym_, and glucoCEST data were analyzed through ROIs on each metabolite tube for the phantom data (Fig. 1A), and on the entire brain, CT and CC of the brain sections matching to those on the autoradiographs (Fig. 2A). The characteristics of the glucoCEST contrast were analyzed for signal distributions examining skewness, kurtosis and variance under different saturation amplitudes. The CNR was derived by^83^:

3 $$CNR=\,\frac{glucoCES{T}_{a}-glucoCES{T}_{b}}{\sqrt{({\sigma }_{a}^{2}-{\sigma }_{b}^{2})/2}}\cdot \frac{1}{\sqrt{TR\cdot NEX}},$$

where gluco*CEST*_*a*_ and gluco*CEST*_*b*_ are the mean intensity obtained from 20 mM glucose and PBS for the phantom data, and from CT and CC for the *in vivo* data; and *σ*_*a*_ and *σ*_*b*_ are their standard deviation (SD), respectively. For the TBI study, four glucoCEST data sets were acquired at two saturation powers (1.5 and 3 μT) and at two saturation durations (1 and 2 s). These glucoCEST-weighted images were compared to the autoradiographs for the sensitivity in detecting cerebral glucose changes following TBI. The autoradiographs were down-sampled to match the image resolution of the glucoCEST-weighted images. Each glucoCEST-weighted images and 2DG autoradiographs were normalized to its dynamic range (mean ± 3 × SD) of the image intensity in order to evaluate the agreement and bias between the two glucose measurements. Data from the external capsule, CC, CT and CPu were quantified for correlation analysis (six ROIs in total). Except for those processed by the aforementioned software, all other imaging data were processed via ImageJ (NIH, Bethesda, MD) and in house Matlab (Mathwork, Natick, MA) developed programs.

### Statistical analysis

Statistical analysis was performed by Welch’s t-test with correction of unequal variances to examine the difference in image contrast between the normal and TBI brains using Prism v6.0c (GraphPad Software, Inc., La Jolla, CA). The significance level (𝛼) was predetermined at 0.05 to reject the null hypothesis. The CoV was determined for the reproducibility of the glucoCEST data obtained from the ROIs in the 20 mM phantom and in CT for the brain. Pearson correlation and Bland-Altman analysis were performed to delineate the possible correlation and agreement/difference between the glucoCEST-weighted images and autoradiographs. Pearson correlation coefficient (*r*) and probability (*p*-value) were reported for the correlation analysis. Bias and 95% confidence interval (CI) were marked for the limits of agreement in the Bland-Altman plots. All data are reported as mean ± SD.

## Electronic supplementary material

Supplementary Information
