# Supplementary material for: On the detection of cerebral metabolic depression in experimental traumatic brain injury using Chemical Exchange Saturation Transfer (CEST)-weighted MRI
Source: Sci Rep. 2018 Jan 12;8:669. doi: 10.1038/s41598-017-19094-z (PMC5766554; doi:10.1038/s41598-017-19094-z)
Supplement: Supplementary file 1 — Supplementary Information [file 41598_2017_19094_MOESM1_ESM.docx]

**Supplementary Information**

**On the detection of cerebral metabolic depression in experimental traumatic brain injury using Chemical Exchange Saturation Transfer (CEST)-weighted MRI**

Tsang-Wei Tu^1,2,3*^, Wael G. Ibrahim^4^, Neekita Jikaria^1,2,^^5^, Jeeva P. Munasinghe^6^, Jacklyn A. Witko^1,2^, Dima A. Hammoud^4^, and Joseph A. Frank^1,7^

^1^Frank Laboratory, Radiology & Imaging Sciences, Clinical Center, National Institutes of Health, Bethesda, MD; ^2^Center for Neuroscience and Regenerative Medicine, Henry Jackson Foundation, Bethesda, MD; ^3^Molecular Imaging Laboratory, Department of Radiology, Howard University, Washington DC; **^4^**Center for Infectious Disease Imaging, Radiology and Imaging Sciences, Clinical Center, National Institutes of Health, Bethesda, MD; ^5^Acute Stroke Research Section, National Institute of Neurological Disorders and Stroke, National Institutes of Health, Bethesda, MD; ^6^Mouse Imaging Facility, National Institute of Neurological Disorders and Stroke, National Institutes of Health, Bethesda, MD; ^7^National Institute of Biomedical Imaging and Bioengineering, National Institutes of Health, Bethesda, MD

***Correspondence to Tsang-Wei Tu, PhD [tut@howard.edu]**

Molecular Imaging Laboratory, Department of Radiology, Howard University

2041 Georgia Ave, NW, Washington DC 20060, USA.

Phone: (202) 865-3742; Fax: (202) 865-3722

A linear relationship (*r*>0.9, *p*<0.01, n=5) was observed between the glucoCEST-weighting image contrast and the glucose concentration when saturation power ≥1μT (Fig. S1A). The magnitude of the glucoCEST-weighted signals increased with increasing saturation duration in glucose phantoms, but no significant increase was seen when using saturation duration >2s (Fig. S1B, C). The glucoCEST-weighted signal increased linearly to the increasing saturation power up to 6μT and became leveled out above 7μT (Fig. S1D). Compared to the signal from 20mM glucose solution, there was a trend that lower glucose concentrations required higher saturation power in order to reach the signal plateaus (i.e., did not continue to increase). The glucoCEST-weighted image contrast had a few interactions with the other metabolites tested in the phantom experiments (Fig. S1E). The glucoCEST data acquired using 6μT and 2s generated superior higher (*p*<0.001, n=5) contrast on the 10mM glucose (17.3±0.7, a.u.) than all other metabolites (MI: 10.8±0.9; Glu: 7.5±0.7; Lac: -0.8±0.2; NAA: 0.6±0.3, a.u.). The histograms of the glucoCEST-weighted signals were approximately Gaussian for glucose (Fig. S2A), with the kurtosis and skewness between -1 and 1 for all the tested saturation amplitudes suggesting the preservation of normality (Fig. S2B). The SDs of the glucoCEST signals increased when using higher saturation power and in higher glucose concentrations (Fig. S2C). The CNRs between glucose and PBS were maximized by applying 5‒7 μT for saturation power and 2‒3 s for saturation duration (Fig. S2D). The CNRs decreased when the saturation power was larger than 7μT.

**
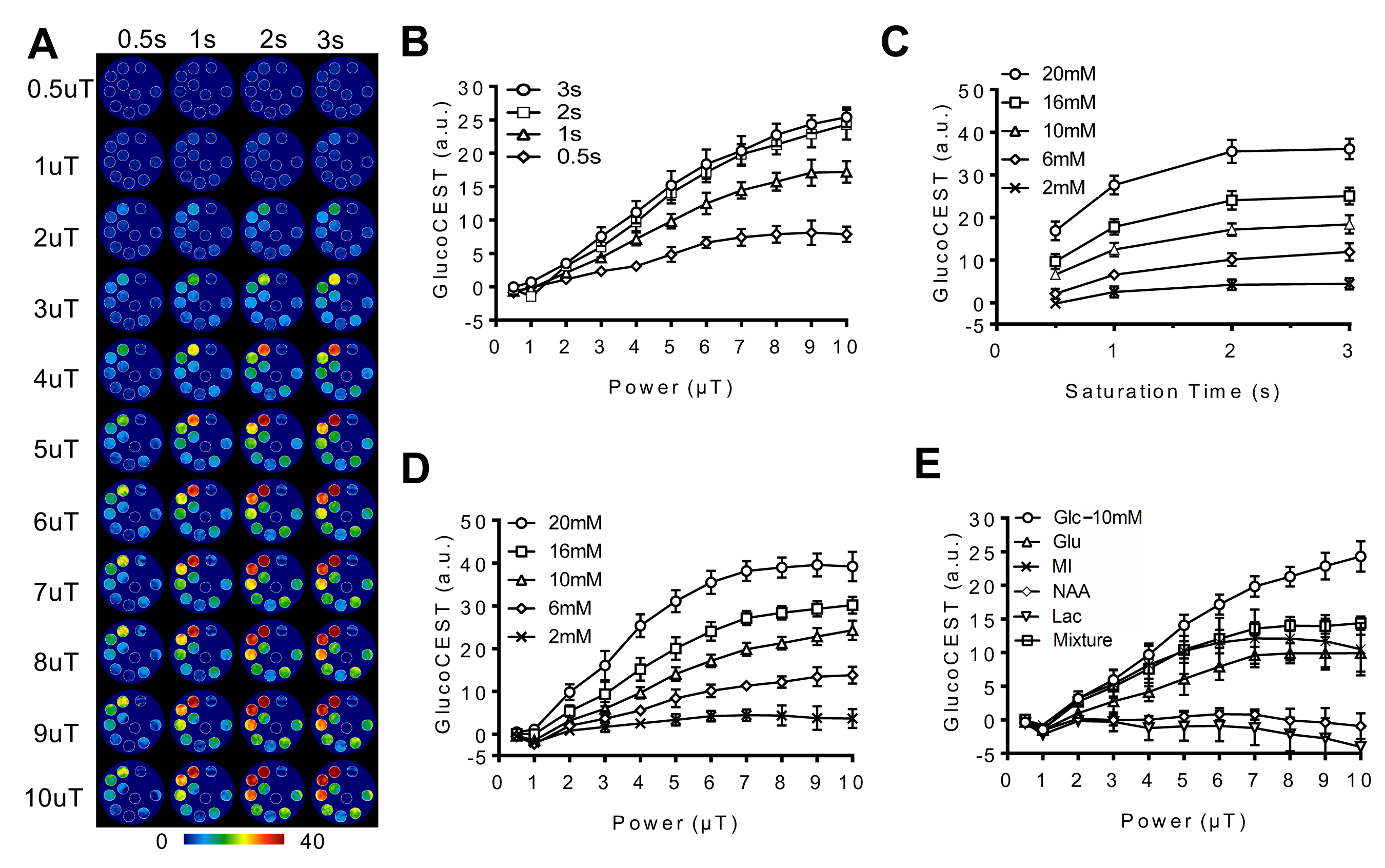
**

**Supplementary Figure S1. Series glucoCEST-weighted images for phantoms** show the contrast changes in response to the array of saturation power and duration (A). The glucoCEST-weighted data acquired from 10mM glucose show the contrast responding to various saturation power and duration (B). No significant difference is seen between the data of 2s and 3s saturation duration regardless of glucose concentrations (C, power fixed at 6μT). The data in (D) illustrates the glucoCEST-weighted contrast changes in response to saturation power (duration fixed at 2s) in glucose phantoms. The data in (E) compare the glucoCEST-weighted contrast reacting to other metabolites in the same acquisition parameters. Glc: glucose, MI: myo-inositol, Glu: glutamate, Lac: lactate, NAA: N-Acetylaspartic acid.

**
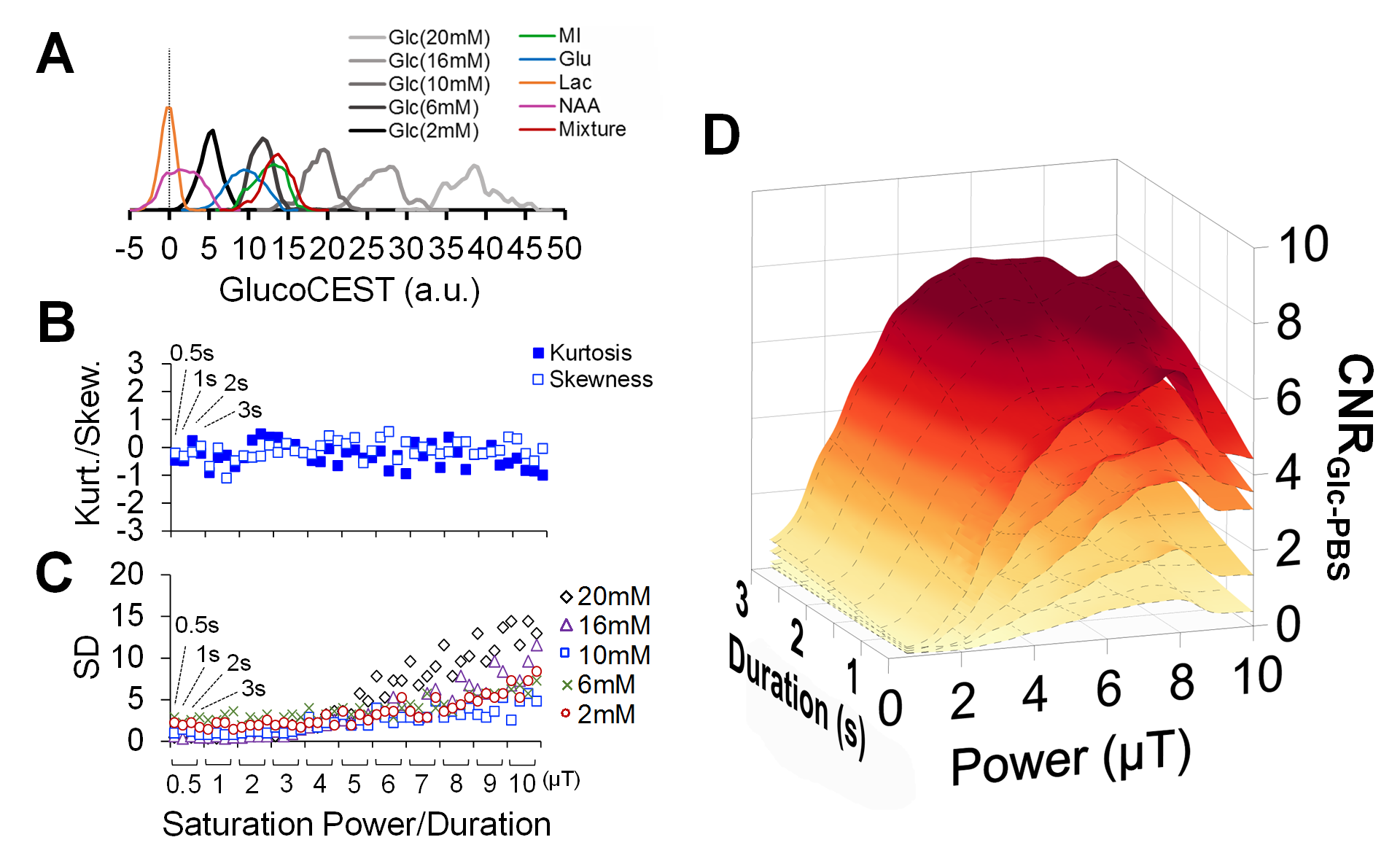
**

**Supplementary Figure S2. The signal distributions of glucoCEST-weighted images** **in the phantom metabolites** using saturation power 6μT and 2s for duration (A). The glucoCEST weighted signals are approximately Gaussian distributed for glucose, where the skewness and kurtosis are roughly maintained at the same level across all saturation amplitudes (B, data acquired from 10mM glucose). The standard deviation (SD) of glucoCEST-weighted image increased when using higher saturation power, and in the phantom of high concentration (C). In B and C, for each saturation power, points from the left to right denote the data acquired by 0.5, 1, 2 and 3s saturation duration. The CNR increased when using higher saturation power in all five glucose concentrations; however, it was not improved when using the saturation power higher than 7μT (D). Glc: glucose, MI: myo-inositol, Glu: glutamate, Lac: lactate, NAA: N-Acetylaspartic acid.
